# Treatment of Peripheral Vascular Graft Infections with Xenogeneic Grafts—A Single-Center Experience

**DOI:** 10.3390/jfb16020048

**Published:** 2025-02-01

**Authors:** Agnieszka Święszek, Wiktor Kruczek, Michał Serafin, Dorota Łyko-Morawska, Tomasz Urbanek, Wacław Kuczmik

**Affiliations:** Department of General Surgery, Vascular Surgery, Angiology and Phlebology, Faculty of Medical Sciences in Katowice, Medical University of Silesia, 45-47 Ziołowa Street, 40-635 Katowice, Poland

**Keywords:** vascular graft infections, No-React^®^ graft, bovine pericardium, xenogeneic graft, infection control, vascular reconstruction, reinfection prevention

## Abstract

**Introduction:** Vascular graft infections (VGEIs) are rare but severe complications in vascular surgery. The choice of reconstruction material following graft removal is critical, particularly for infection prevention. This study evaluates the use of No-React^®^ BioIntegral Surgical Grafts, made from bovine pericardium, in the treatment of VGEIs. **Materials and Methods:** A retrospective study of 12 patients (mean age 66.5 years; 67% male) treated between 2020 and 2022 was conducted. The follow-up period included in the study extended from the date of the procedure to 30 June 2024. **Results:** The study observed a 0% reinfection rate, underscoring the anti-infective potential of No-React^®^ grafts. However, in-hospital complications were frequent, affecting six (50%) patients, with sepsis (3; 25%) related to preoperative VGEIs being the most common. Most importantly, in-hospital mortality was notably high (42%), primarily driven by infection-related sepsis. The overall complication rate after discharge was 14%, with only one case of graft occlusion (1/7) observed. Among discharged patients (7; 58%), the three-month survival rate was 71%. In-hospital complications were a predictive factor for overall survival (OS) (HR = 15.88, 95% CI = 1.81–139.47). **Conclusions:** Xenogeneic No-React^®^ grafts show promise for managing VGEIs, offering low reinfection rates. However, high morbidity and mortality underline the challenges of treating patients with severe VGEIs. Early postoperative complications were a key predictor of OS. Further research is needed to confirm these findings and optimize treatment protocols for VGEIs.

## 1. Introduction

Cardiovascular diseases are widely recognized as one of the leading causes of death globally. The increasing prevalence of life-threatening conditions such as occlusive vascular disease, aortic aneurysms, and aortic dissections has led to a rising demand for vascular procedures. Peripheral artery disease necessitates interventions to manage ischemia, aiming to prevent limb amputation and profound disability [1]. Therefore, the utilization of synthetic vascular prostheses has surged, paralleling the growing number of patients undergoing vascular surgery procedures. Unfortunately, this has also contributed to a rise in graft-related infections. Vascular graft or endograft infections (VGEIs) remain rare but severe complications following vascular surgery interventions. Research from Pilsen, Czech Republic, estimates that 1% to 6% of patients undergoing peripheral vessel reconstruction and 0.5% to 4% of those undergoing aortic reconstruction may be affected by graft infections [2].

The morbidity and mortality rates associated with VGEIs are notably high. In the aforementioned study, mortality rates reached up to 40%, varying based on the site of vascular reconstruction and the severity of vascular prosthesis infection [2]. Assessing the true prevalence of VGEIs is challenging due to their multifactorial nature, influenced by surgical techniques, environmental factors, and individual patient characteristics [3].

Infections related to vascular grafts can be classified based on the extent of graft involvement. Moreover, VGEIs can be further categorized into early-onset infections (occurring within four months of graft placement) and late-onset infections (occurring over four months after placement). One presumption regarding early infections is that they are primarily caused by sterility breaches during implantation or by pre-existing bacterial presence in the aneurysmal thrombus [4]. On the other hand, late infections are believed to result from bacteremia originating from the respiratory or urinary tract. Another potential cause is bacterial translocation stemming from iatrogenic contamination, such as dental procedures (e.g., tooth extraction). The spectrum of clinical manifestations of graft infection is influenced by the virulence of the infectious organism. Patients face potential threats to their lives from both sepsis and massive bleeding in the event of vascular anastomosis loosening [4,5,6].

The fundamental principles of managing VGEIs involve the removal of the infected prosthesis, revascularization, and supportive antimicrobial therapy. Infected synthetic grafts serve as peculiar bacterial culture media, fostering bacterial accumulation and facilitating infection spread. However, determining the most suitable material for implantation in place of a previously infected synthetic vascular graft remains a challenge. Options such as autologous veins, cryopreserved allografts, rifampicin-bonded grafts, or silver-coated grafts have been explored [1,4]. A recent development in this field is the emergence of fully biological grafts made from bovine pericardium, such as the No-React^®^ BioIntegral Surgical Graft [1].

The aim of this study was to evaluate the safety and efficacy of No-React^®^ BioIntegral Surgical Graft implantation as a treatment option for vascular VGEIs in patients with previously infected artificial or autologous grafts. The study also sought to analyze clinical features, microbial profiles, and postoperative outcomes of patients with VGEIs.

## 2. Materials and Methods

### 2.1. Study Design and Population

We retrospectively analyzed the medical records of patients treated with peripheral bypass and included only patients who underwent surgical treatment for graft infections using the No-React^®^ nonvalved conduit (Biointegral Surgical Inc., Mississauga, ON, Canada) from January 2020 to December 2022 in the Department of General Surgery, Vascular Surgery, Angiology, and Phlebology, Medical University of Silesia in Katowice, Poland. Additionally, patients underwent follow-up evaluations at the hospital’s surgical clinic. The follow-up period for the study extended from the date of the procedure to 30 June 2024.

Between January 2020 and December 2022, 389 patients underwent peripheral bypass procedures, including 12 (3.08%) patients treated with the No-React^®^ nonvalved conduit. 

Therefore, the study group of this retrospective analysis consisted of 12 adult patients (8 men, 67%; 4 women, 33%) with a mean age of 66.50 years (range: 56–77, standard deviation (SD) = 5.81). 

### 2.2. Inclusion/Exclusion Criteria for the Study and Surgical Treatment 

For the study population, the inclusion criteria were defined as cases of synthetic or autologous graft infections treated specifically with xenogeneic prostheses. The exclusion criteria were VGEIs treated with autologous, synthetic, or homologous grafts.

The inclusion criteria for the surgical procedures included cases of VGEIs where suitable patients’ native veins were unavailable for reconstruction. The exclusion criteria consisted of the presence of suitable native veins and lack of patient consent for the procedure.

### 2.3. Analyzed Data

Parameters such as patients’ general characteristics (age, gender, comorbidities, and clinical symptoms), type and duration of surgery, incidence of postoperative complications, reoperations, mortality, duration of hospitalization, as well as preoperative blood test results, blood culture, intraoperative synthetic prosthesis culture, and follow-up were analyzed in the study.

### 2.4. Biological Prosthesis

The No-React^®^ nonvalved conduit is an on-shelf available vascular graft that derives from bovine pericardium crosslinked with glutaraldehyde and benefits from a proprietary surface treatment involving heparin rinsing, which enhances its biocompatibility by sealing the graft’s surface. This process supports endothelialization, a critical biological mechanism wherein endothelial cells migrate to, adhere to, and proliferate on the graft’s surface. Over time, this process leads to the formation of a continuous endothelial layer that mimics the native vascular lining [4,6,7,8,9,10].

Endothelialization is pivotal in reducing the risk of infection because it creates a biological barrier that prevents the direct exposure of the graft material to bloodborne pathogens and inflammatory cells. This layer minimizes bacterial adhesion—a primary step in biofilm formation where microbes colonize a surface and develop a resistant protective matrix. Without endothelial coverage, synthetic or non-endothelialized grafts are more prone to biofilm development, which is notoriously difficult to eradicate with antibiotics [4,6,7,8,9,10].

The biological composition of the No-React^®^ conduit further supports endothelialization by reducing the inflammatory response that synthetic materials often provoke. Unlike synthetic grafts, bovine pericardium provides a collagen-rich matrix that closely resembles native tissue, offering an ideal substrate for cellular adherence and integration. These properties make the No-React^®^ conduit particularly advantageous for use in patients at a heightened risk of infection, such as those with pre-existing graft infections, especially in the absence of the patient’s autologous material and/or in emergency settings where the patient would not tolerate the harvesting of autologous material [4,6,7,8,9,10].

### 2.5. Surgical Procedure

The surgical intervention involved the excision of infected tissues and removal of the synthetic graft, followed by local debridement to achieve optimal wound-bed preparation. For vascular reconstruction, a bovine pericardial graft was utilized, with dimensions precisely tailored on a 1:1 basis to match the native vessel diameter of each patient, ensuring anatomical compatibility and functional integrity. Postoperative management included an intensive in-hospital regimen of intravenous antimicrobial therapy targeted to the pathogen profile obtained through preoperative and intraoperative microbiological analyses. Upon discharge, patients were transitioned to an extended course of oral antimicrobial agents to ensure the eradication of residual infection and reduce the risk of recurrence.

### 2.6. Statistical Analysis 

The statistical analysis was performed using Statistica^®^ (New York, NY, USA, 2013) software version 13.3 (StatSoft). Absolute values and percentages were used to present qualitative variables, and ranges, means, and standard deviations or medians with interquartile ranges were applied for quantitative variables. The Shapiro–Wilk test was used to determine the statistical distribution among quantitative variables. Overall survival analysis was performed using the Kaplan–Meier estimator. The analysis of prognostic factors was performed using univariate Cox proportional hazards. A *p*-value < 0.05 was considered statistically significant.

## 3. Results

### 3.1. Patients’ General Characteristics

All patients (100%) presented comorbidities, with arterial hypertension, general atherosclerosis, and coronary artery disease being the most common, observed in nine (75%) patients for each condition. Additionally, nine (75%) patients had a history of cigarette smoking. Chronic limb ischemia was identified as the primary cause of vascular prosthesis implantations in all 12 (100%) patients. The predominant primary surgical treatment was aorto-bifemoral bypass, conducted in 7 (58%) patients. The median time between the primary procedure and VGEI diagnosis was 45 (6–97) months. Upon admission, clinical symptoms were evident in 10 (83.33%) patients, with purulent infection sites being the most frequently observed (9; 75%) (Table 1). 

### 3.2. Blood Test Results

In the preoperative blood tests, seven (58%) patients had elevated white blood cell (WBC) counts. Eight (67%) patients had low hemoglobin levels. Furthermore, the mean C-reactive protein level was elevated in 12 (100%) patients. Glucose levels averaged 107.00 (92.00–311.00) mg/dL, IQR 94 mg/dL (Table 2).

### 3.3. Surgical Characteristics

Most patients (6; 50%) were assessed in the fourth group of the American Society of Anesthesiologists (ASA) scale. The mean duration of the procedure was 282.33 (143–400), SD 93.91 min. The most common type of bypass performed was aorto-femoral bypass in six (50%) patients. The median duration of hospitalization was 18.5 (12–114), IQR 27 days, and the duration of hospitalization after the procedure was 8 (2–110), IQR 10 days. 

In-hospital complications occurred in six (50%) patients, with the most common being sepsis (3; 25%). In-hospital reoperation was required in four (33%) patients due to colon perforation (2; 17%), acute limb ischemia (1; 8%), and femoral hematoma (1; 8%). Five (42%) deaths occurred during postoperative hospitalization due to sepsis (3; 25%) related to preoperative graft infection and multiorgan failure related to ischemic colon and colon perforation (2; 17%). Detailed intraoperative characteristics and complications are presented in Table 3.

### 3.4. Pre- and Intraoperative Culture and Antibiotic Therapy

All patients before surgical treatment had a bacteriological culture taken from the infection site and blood. The most common bacteria present on infection sites was Staphylococcus aureus (*S. aureus*) (4; 33%) with methicillin resistance (MRSA) (3; 25%). Also, in blood cultures, the most commonly observed bacteria was *S. aureus* (3; 25%) with methicillin resistance (MRSA) (3; 25%). In addition, after the surgical removal, all grafts were sent for bacteriological culture. The most common bacteria isolated from the prosthesis culture was Staphylococcus epidermidis (*S. epidermidis*) (3; 25%) with methicillin resistance (MRSA) (3; 25%). Throughout their hospitalization, all patients received antibiotic therapy. Detailed information on bacteriological cultures and antibiotic therapy is provided in Table 4.

### 3.5. Follow-Up

The median follow-up was 96 (2-1001), IQR 943 days with in-hospital deceased patients and 915 (55-1001), IQR 1127 days in patients discharged from the hospital after surgical procedure. In the follow-up period, one (14%) graft occlusion occurred out of seven discharged patients. In addition, two (29%) out of seven discharged patients died due to COVID-19. Overall, the three-month survival was 50%, standard error = 15%. (SE 17%) (Table 5).

The univariate Cox proportional hazards regression model analysis showed that the occurrence of in-hospital complications (hazard ratio (HR) = 15.88; 95% confidence interval (CI) = 1.81–139.47; *p* = 0.01) was the only predictive factor for overall survival (OS) (Table 6). 

## 4. Discussion

The clinical manifestations of VGEIs are typically conspicuous. Recent research indicates that a majority of patients exhibit symptoms either systemically or related to the site of infection. Predominant systemic symptoms include elevated levels of C-reactive protein (>5 mg/L), observed in 71% to 100% of patients, and elevated white blood cell (WBC) counts (>10 thousand/μL) in 61–75% of patients. Conversely, the most prevalent site-specific symptoms include pain reported in 75–88% of cases and purulent manifestations at the infection site in 65–88% [1,11]. In our cohort, all patients exhibited elevated C-reactive protein levels, with an additional 58% of patients demonstrating increased WBC counts. Notably, our study identified purulent infection sites in 75% of cases, while pain was reported in 42% of patients. These findings underscore the importance of promptly investigating local symptoms and excluding prosthesis-related infection when elevated inflammatory markers are detected among patients with vascular prostheses.

Fighting an artificial prosthesis infection often parallels military tactics. Hence, the principle of understanding one’s adversary, crucial in warfare, can be extrapolated to prosthetic infections. Recent studies indicate that Gram-positive bacteria, MSSA, MRSA, and coagulase-negative staphylococci (CoNS) are responsible for a significant portion of VGEIs, ranging from 34% to 75% [12,13,14]. Among our patients, MRSA was detected in three cases (25%) in the site culture, MSSA in one case (8%), and MSSE in one case (8%), constituting 42% of all cultures collected. Similarly, in prosthesis cultures, MRSE was identified in three cases (25%), while MRSA was found in two cases (17%), making up 42% of all infected prostheses. According to ESVS guidelines, treatment with broad-spectrum antibiotics should be initiated as soon as possible if a prosthetic infection is suspected. Therefore, based on the above results, empirically used antibiotics should target gram-positive bacteria, including resistant strains (such as vancomycin and ciprofloxacin). Subsequently, treatment should be adjusted according to the results of collected bacteriological cultures [4].

In the surgical treatment of VGEIs, several methods can be used, including in situ reconstruction (ISR) with prosthetic grafts, cryopreserved allografts, and autologous material [1,4,15].

In situ reconstruction with prosthetic grafts is often considered safe, with short operative times. Additionally, these grafts are readily available for use. In this approach, prosthetic grafts such as silver-impregnated or rifampicin-soaked polyethylene terephthalate (PET) grafts are utilized. The morbidity associated with prosthetic grafts for VGEI reconstruction typically ranges between 42.2% and 78%, while 30-day mortality rates vary between 19.7% and 25%. Reintervention rates differ between 14% and 30% [15,16,17]. However, the major drawback of this method is the high reinfection rate, which can be as high as 19%. Recent data also indicate that in up to 31% of VGEI cases, the microorganism responsible for the infection may exhibit resistance to rifampicin [18,19].

The second ISR method involves the use of cryopreserved allografts. This approach exhibits reinfection rates ranging from 6% to 16%, which are lower than those observed with prosthetic grafts. The morbidity associated with this method ranges between 50.7% and 70%, while 30-day mortality varies from 6% to 30% [15,20,21]. However, the major drawback of this method is the high five-year reintervention rate, ranging from 33% to 50%, due to graft degeneration [15,22].

The most promising option for ISR involves utilizing autologous graft materials such as the great saphenous or femoral vein [23,24]. Reinfection rates are notably low, ranging between 0% and 6.45% [25,26]. Additionally, morbidity varies between 55% and 61%, while 30-day mortality rates range from 6.6% to 17% [15,25,26]. Moreover, reintervention rates can be as high as 43%. The primary limitation of this approach lies in its reliance on the availability of the patient’s native veins, which may not always be feasible. Consequently, alternative materials for ISR in such patients must be considered [22].

One of the newest materials used for ISR is xenogeneic grafts made from bovine pericardium, specifically The No-React^®^ conduit from Biointegral Surgical Inc. These grafts have been employed for several decades in the treatment of endocarditis. Studies conducted on endocarditis treatment have indicated that xenogeneic grafts serve as a viable option when a patient’s native veins are unavailable. The primary factors influencing patient outcomes include the timing of treatment initiation, particularly in emergency cases. Patients treated electively have demonstrated superior outcomes compared to emergency cases [1]. However, to date, only a limited number of studies have been conducted on the No-React^®^ xenogeneic grafts in the treatment of VGEIs. The largest study to date was conducted in the Netherlands by Folmer et al. and involved a group of 34 patients [1]. In this study, the reinfection rate was 9%, with a morbidity rate of 29% and a 30-day mortality rate of 12%. Notably, the rate of reinterventions was 8.82%. In our study, we observed a reinfection rate of 0%. Importantly, the 30-day mortality rate was 42%, with surgery-related mortality accounting for 17%. Additionally, the morbidity rate was 50%, with surgery-related complications occurring in 25% of cases. The rate of reinterventions within 30 days was 33%, with an overall rate of 50%. Based on these findings, it can be concluded that reinfection rates in xenogeneic grafts are comparable to those of autologous grafts. The mortality and morbidity rates observed in our study suggest that the No-React^®^ grafts can be compared to prosthetic grafts. However, our results notably exceed those reported in the study by Folmer et al. This disparity may be attributed to the severe condition of our patients and a higher proportion of emergency procedures. Specifically, half of our patients were classified as ASA IV, while two were classified as ASA V. Furthermore, seven of our patients underwent emergency procedures.

The methods of surgical intervention for VGEIs have their respective advantages and disadvantages, but how do they compare to conservative treatment? In a study conducted by Seleem et al., the five-year mortality rate among patients treated conservatively with observation and antibiotics was reported to be 45%. Furthermore, conservative treatment was identified as an independent predictive factor for mortality, with a hazard ratio of 3.62 [27].

When examining long-term outcomes in patients treated with various ISR methods, for prosthetic grafts, the five-year mortality rate is estimated to be around 20% [15]. For cryopreserved allografts, the five-year mortality rate can be up to 35% [15,20]. For autologous graft ISR, the mortality rates range between 25% and 40% [15,28]. For the No-React^®^ xenogeneic grafts, in the study by Folmer et al., the mortality rate was reported to be 30% [1].

In our study, the overall three-month mortality rate was 50%. It is noteworthy that the majority of patients (5; 42%) in our study died in the hospital after the surgical procedure. Additionally, among patients who were discharged from the hospital (7; 58%), two (29%) deaths were observed. The in-hospital mortality rate of 42% and the 50% complication rate raise concerns about the safety of the intervention. However, these outcomes are likely reflective of the critical condition of the patients prior to surgery. Notably, eight patients were classified as ASA grade IV or V, and seven patients underwent emergency procedures. Furthermore, three out of five in-hospital deaths were directly caused by sepsis associated with preoperative prosthetic graft infection. It is also important to highlight that all deaths among discharged patients were related to COVID-19 infection rather than complications associated with the surgical intervention itself or the No-React^®^ grafts. Nonetheless, the overall findings suggest that the survival rate among patients undergoing surgery is lower compared to those treated conservatively. While the overall mortality rates reported in our study are concerning, it is worth emphasizing that the mortality rates among patients discharged from our department are notably lower than the five-year mortality rates reported for conservative treatment, as shown in the study by Seleem et al. Most deaths in our study occurred within the first 30 days after the surgical procedure, with most of them related to preoperative, severe patient conditions, underscoring the critical nature of the patient’s condition in VGEIs and the early postoperative period. Despite the challenges and risks associated with surgery, it appears to offer a better chance of survival compared to conservative management in cases of VGEIs.

The predictive factor for OS in our study was observed to be the occurrence of in-hospital complications (HR = 15.88, 95% CI = 1.81–139.47; *p* = 0.01). This finding suggests that in-hospital complications can increase the risk of death in patients undergoing surgical treatment of VGEIs by almost 16 times. However, the confidence interval is notably wide, likely due to the small sample size of our study. Despite this limitation, our data align with the existing literature on vascular procedures, which consistently identifies the occurrence of complications as a significant predictive factor for mortality. In their study, Varkevisser et al. observed that the occurrence of complications has a significant impact on mortality, with HR = 5.9; 95% CI = 3.9–9.1; *p* < 0.001 [29]. This association may stem from the reality that patients with vascular disease, particularly those with VGEIs, frequently present in a severe condition (e.g., eight of the patients in our study were assessed to ASA groups IV or V), and the occurrence of complications decreases their likelihood of survival. Nevertheless, the small sample size may have influenced our results, underscoring the need for larger studies focused on identifying predictive factors for OS in patients undergoing surgical treatment for VGEIs.

### Study Limitations

There are several significant limitations to this study that must be acknowledged. Firstly, the sample size is notably small and restricted, which not only reduces the statistical power of the analysis but also limits the reliability of the results and their applicability to larger or more diverse populations. This small cohort size poses challenges in identifying less frequent but clinically significant outcomes, which may have influenced the overall conclusions. Secondly, the retrospective nature of the study, conducted within a single medical center, introduces several constraints. Retrospective analyses inherently rely on the accuracy and completeness of pre-existing medical records, which can vary in detail and quality. Additionally, the retrospective design increases the likelihood of selection bias and limits the ability to account for all confounding variables, making causal relationships difficult to establish. Furthermore, the single-center setting may reflect local practices and patient demographics that are not representative of broader populations, further restricting the generalizability of the findings. Lastly, the study was conducted during the COVID-19 pandemic, specifically between 2020 and 2022, a period marked by significant disruptions in healthcare systems. Delays in patient diagnoses and treatments during this time may have contributed to a higher prevalence of severe and emergent cases in the study population. As a result, the reported morbidity and mortality rates may not accurately reflect those observed under typical clinical conditions, potentially leading to an overestimation of the severity of VGEIs in this context. Despite these limitations, this study includes one of the largest cohorts of patients analyzed for VGEI treatment with xenografts. To the best of the authors’ knowledge, it represents the second-largest study overall and the largest single-center analysis of this specific treatment modality. Future research should aim to address these limitations by including larger, multi-center cohorts and prospectively evaluating a broader range of treatment strategies for VGEIs, encompassing both invasive and conservative approaches.

## 5. Conclusions

Our study examined the clinical features and microbial profile of VGEIs. Xenogeneic grafts, such as the No-React^®^ conduit, offer a new option for the treatment of VGEIs; however, further research is required. The xenograft demonstrated low reinfection and low reintervention rates. Early postoperative complications were a predictive factor for overall survival (OS), emphasizing the critical nature of this period. Timely diagnosis, appropriate antibiotic use, and tailored surgical intervention are essential in managing VGEIs. Further research is needed to refine treatment approaches and improve outcomes for this challenging patient group.

## Figures and Tables

**Table 1:** General characteristics of patients.

Patient Number	Patient Age (y)	Body Mass Index	Symptoms	Comorbidities	Drugs	Medications	Primary Procedure	Time After Primary Procedure to VGEI (Months)
1	64	16.02	Groin pain	Arterial hypertension, generalized atherosclerosis, coronary artery disease, heart failure, diabetes mellitus	Cigarettes	Antiplatelet	Aorto-bifemoral bypass with synthetic graft	12
2	62	26.12	Purulent infection site	Arterial hypertension, generalized atherosclerosis, coronary artery disease, heart failure, history of myocardial infarction	No	Antiplatelet, B-blockers, ACEI, anticoagulant	Aorto-bifemoral bypass with synthetic graft	97
3	77	28.34	Purulent infection site	Arterial hypertension, generalized atherosclerosis, coronary artery disease, diabetes mellitus	Cigarettes	Antiplatelet, B-blockers, ACEI, statins	Femoro-femoral bypass with synthetic graft	74
4	69	25.48	Purulent infection site	Arterial hypertensiongeneralized atherosclerosis, coronary artery disease, heart failure, history of myocardial infarction	Cigarettes	Antiplatelet, B-blockers	Aorto-bifemoral bypass with synthetic graft	6
5	72	24.84	No	Arterial hypertension	Cigarettes	Antiplatelet, B-blockers, ACEI	Aorto-bifemoral bypass with great saphenous vein	62
6	56	22.32	Groin pain	Arterial hypertension, generalized atherosclerosis, coronary artery disease	Cigarettes	Antiplatelet, B-blockers, ACEI	Aorto-bifemoral bypass with synthetic graft	68
7	68	29.22	Groin pain, Purulent infection site	Arterial hypertension, generalized atherosclerosis, coronary artery disease, heart failure, diabetes mellitus, COPD	No	Antiplatelet, B-blockers	Aorto-bifemoral bypass with synthetic graft	26
8	70	22.84	Purulent infection site	Arterial hypertension, generalized atherosclerosis, coronary artery disease, history of myocardial infarction	Cigarettes	Antiplatelet, B-blockers, ACEI, anticoagulant, statins	Aorto-bifemoral bypass with synthetic graft	52
9	69	17.31	Groin pain, purulent infection site	Arterial hypertension	Cigarettes	Antiplatelet, anticoagulant	Aorto-femoral bypass with synthetic graft	85
10	61	22.26	Groin pain, purulent infection site	Generalized atherosclerosis, coronary artery disease, history of myocardial infarction, COPD	Cigarettes	Antiplatelet, B-blockers	Aorto-femoral bypass with synthetic graft	37
11	69	15.63	Purulent infection site	Heart failure, history of myocardial infarction,	Cigarettes	Antiplatelet, anticoagulant, statins	Femoro-femoral bypass with synthetic graft	30
12	61	22.27	Purulent infection site	Generalized atherosclerosis, coronary artery disease, COPD	Cigarettes	Antiplatelet	Aorto-femoral bypass with synthetic graft	30

Abbreviations: ACEI—Angiotensin Converting Enzyme Inhibitor, COPD—Chronic obstructive pulmonary disease.

**Table 2:** Preoperative blood test results.

Variable	n (%); Mean/Median (Range, SD/IQR)
White blood counts (thousand/μL)	10.81 (4.95–16.10), SD 3.67
Hemoglobin (g/dL)	11.82 (9.70–16.50), SD 1.87
Hematocrit (%)	35.06 (29.20–47.10), SD 4.95
Platelets (tys./μL)	243.58 (148–436), SD 81.74
Neutrophils (thousand/μL)	7.42 (4.69–11.35), SD 2.17
Lymphocytes (thousand/μL)	1.85 (0.57–3.76), SD 0.88
C-reactive protein (mg/L)	66.88 (7.00–132.00), SD 56.02
Procalcitonin (ng/mL)	0.09 (0.03–0.06), IQR 0.06
Potassium (mmol/L)	4.07 (3.40–4.60), SD 0.41
Sodium (mmol/L)	139.00 (125.00–143.00), IQR 4
Glucose (mg/dL)	107.00 (92.00–311.00), IQR 94

Abbreviations: SD—Standard Deviation, IQR—Interquartile range.

**Table 3:** Patients’ surgical characteristics.

Patient No.	ASA	Duration of Procedure (Minutes)	Bypass Type	Intraoperative Blood Loss (mL)	Transfusion of Red Blood Cells (RBC)	Transfusion of Fresh Frozen Plasma (FFP)	Duration of Hospitalization (Days)	Duration of Postoperative Hospitalization (Days)	In-Hospital Complications	In-Hospital Reoperation	In-Hospital Mortality
1	III	235	Aorto-bifemoral	800	Yes	No	41	3	Acute limb ischemia, sepsis	Surgical thrombectomy	Yes
2	V	397	Aorto-femoral	1500	Yes	Yes	114	110	Colon perforation	Colostomy	Yes
3	III	345	Femoro-femoral	<400	No	No	13	4	No	No	No
4	IV	370	Aorto-femoral	650	Yes	No	30	12	No	No	No
5	III	400	Aorto-bifemoral	700	Yes	No	12	2	Sepsis	No	Yes
6	IV	303	Aorto-bifemoral	850	Yes	No	23	19	No	No	No
7	V	350	Aorto-bifemoral	<400	No	No	39	35	Sepsis	No	Yes
8	IV	180	Aorto-femoral	500	Yes	No	14	9	No	No	No
9	IV	290	Aorto-femoral	<400	No	No	44	11	Colon perforation	Colostomy	Yes
10	IV	143	Aorto-femoral	<400	No	No	13	7	No	No	No
11	III	225	Femoro-femoral	<400	No	No	13	7	No	No	No
12	IV	150	Aorto-femoral	<400	No	No	13	7	Hematoma	Surgical drainage	No

Abbreviations: ASA—American Society of Anesthesiologists.

**Table 4:** Bacteriological culture results and antibiotic therapy.

Patient No.	Groin Skin Culture	Groin Skin Culture Resistance	Blood Culture	Blood Culture Resistance	Graft Culture	Graft Culture Resistance	In-Hospital Antibiotic Therapy	In-Hospital Antibiotic Therapy (Days)	Discharge Antibiotic Therapy
1	*S. aureus*	MSSA	*-*	*-*	*S. epidermidis*	MRSE	Amoxicillin + clavulanic acid, ciprofloxacin, vancomycin, clindamycin, gentamicin, metronidazole.	41	*-*
2	*Ent. faecalis*	VRE	*-*	*-*	-	-	Ampicillin + sulbactam, Amoxicillin + clavulanic acid, ciprofloxacin, meropenem, ceftriaxone, sulfamethoxazole + trimethoprim, colistin, vancomycin, metronidazole.	114	Colistin
3	*S. epidermidis*	MSSE	*-*	*-*	*S. epidermidis*	MRSE	Ciprofloxacin, sulfamethoxazole + trimethoprim, vancomycin.	8	Amoxicillin + clavulanic acid,
4	*A. baumanii*	ESBL	*-*	*-*	-	-	Cloxacillin, Meropenem, Colistin, Gentamicin, Metronidazole.	17	Doxycycline
5	*S. aureus*	MRSA	*-*	*-*	-	-	Ciprofloxacin, Vancomycin.	12	*-*
6	*Klebsiella pneumoniae*	ESBL	*Ent. cloacae*	ESBL	*Ent. cloacae*, candida guilliermondii	ESBL	Amoxicillin + clavulanic acid, ciprofloxacin, meropenem, vancomycin, clindamycin.	41	Ciprofloxacin
7	*Klebsiella pneumoniae*	ESBL	*S. aureus*	MRSA	-	-	Cloxacillin, Ciprofloxacin, Meropenem, Linezolid, Vancomycin.	39	Ciprofloxacin
8	-	-	*-*	*-*	-	-	Vancomycin	13	Ciprofloxacin
9	Ent. faecalis	VRE	*-*	*-*	*S. epidermidis*	MRSE	Ampicillin + sulbactam, amikacin, tigecycline, Meropenem, linezolid, Vancomycin, clindamycin.	48	*-*
10	*S. aureus*	MRSA	*S. aureus*	MRSA	*S. aureus*	MRSA	Vancomycin,	12	*-*
11	*-*	*-*	*-*	*-*	Finegoldia magna	ESBL	Sulfamethoxazole + trimethoprim, clindamycin.	12	Clindamycin
12	*S. aureus*	MRSA	*S. aureus*	MRSA	*S. aureus*	MRSA	Vancomycin,	20	*-*

Abbreviations: *S. aureus*—*Staphylococcus aureus*, *S. epidermidis*—*Staphylococcus epidermidis*, *Ent. faecalis*—*Enterococcus faecalis*, *Ent. cloacae*—*Enterococcus cloacae*, *A. baumanii*—*Acinetobacter baumannii*, MRSA—Methicillin-resistant Staphylococcus aureus, MSSA—Methicillin-sensitive Staphylococcus aureus, MRSE—Methicillin-resistant Staphylococcus.

**Table 5:** Follow-up data.

Patient No.	Follow-Up Time (Months)	Complications	Reoperations	Reinfection	Alive at 3 Months	Cause of Death
3	41	No	No	No	Yes	-
4	40	Graft occlusion	Mechanical thrombectomy	No	Yes	-
6	35	No	No	No	Yes	-
8	30	No	No	No	Yes	-
10	2.5	No	No	No	No	COVID-19
11	28	No	No	No	Yes	-
12	2	No	No	No	No	COVID-19

**Table 6:** Univariate analysis of factors with mortality using Cox proportional hazards regression model.

Variate	Survival Time (Months)	HR	95% CI	*p* (df = 1)
Age		0.94	0.82–1.08	0.41
BMI		0.96	0.81–1.14	0.14
Gender				
Male	3.2 (1.117–33.37) IQR 19.54	0.30	0.06–1.4	0.12
Female	0.6 (0.07–20.42) IQR 10.68	1		
History of cigarette smoking				
Yes	1.83 (0.07–33.37), IQR 19.33	3.35	0.39–28.32	0.21
No	22.07 (3.67- 32.7) IQR 29.03	1		
Arterial hypertension				
Yes	3.67 (0.07–33.37), IQR 24.93	0.99	0.49–5.17	0.99
No	2.73 (1.83–20.43), IQR 18.60	1		
General atherosclerosis				
Yes	3.67 (0.1–33.37), IQR 24.20	0.54	0.1–2.82	0.47
No	1.1 (0.07–20.43), IQR 20.36	1		
Coronary artery disease				
Yes	3.67 (0.1–33.37), IQR 24.2	0.54	0.1–2.82	0.47
No	1.1 (0.07–20.43), IQR 20.36	1		
History of myocardial infarction				
Yes	12.87 (2.73–32.7), IQR 24.19	0.48	0.08–2.19	0.29
No	1.50 (0.07–33.37) IQR 22.63	1		
Diabetes mellitus				
Yes	1.17 (0.1–33.7), IQR 33.27	0.65	0.13–3.39	0.61
No	3.67 (0.07–32.7), IQR 20.24	1		
Heart failure				
Yes	2.42 (0.1–32.7), IQR 17.55	1.48	0.33–6.67	0.60
No	11.58 (0.07–33.37) IQR 22.59	1		
History of stroke				
Yes	1.17 (1.1–3.67), IQR 2.57	2.68	0.59–12.19	0.20
No	20.43 (0.07–33.37) IQR 24.20	1		
COPD				
Yes	1.83 (1.17–2.73), IQR 1.56	2.77	0.55–13.99	0.22
No	20.43 (0.07–33.37), IQR 24.93	1		
Blood test results				
White blood counts (thousand/μL)		1.17	0.93–1.46	0.18
Hemoglobin (g/dL)		1.09	0.69–1.74	0.71
Hematocrit (%)		1.03	0.87–1.23	0.71
Platelets (tys./μL)		1	0.99–1.01	0.73
Neutrophils (thousand/μL)		1.17	0.72–1.89	0.51
Lymphocytes (thousand/μL)		0.71	0.22–2.24	0.56
C-reactive protein (mg/L)		1	0.98–1.02	0.72
Procalcitonin (ng/mL)		0.89	0.76–1.07	0.22
Potassium (mmol/L)		1.02	0.12–8.66	0.98
Sodium (mmol/L)		1	0.99–1.1	0.35
In-hospital complications				
Yes	1.14 (0.07–3.67), IQR 1.73	15.88	1.81–139.47	0.01
No	24.05 (2.73–33.37), IQR 12.27	1		

Abbreviations: IQR—Interquartile range, HR—hazard ratio, 95% CI—95 % Confidence Interval, BMI—Body Mass Index, COPD—chronic obstructive pulmonary disease.

## Data Availability

The original contributions presented in the study are included in the article, further inquiries can be directed to the corresponding authors.
